# Midday meals as an early childhood nutrition intervention: evidence from plantation communities in Sri Lanka

**DOI:** 10.1186/s12889-021-11843-0

**Published:** 2021-12-07

**Authors:** Udeni De Silva Perera, Brett A. Inder

**Affiliations:** 1 Centre for Health Economics, Monash Business School, Monash University, Melbourne, Australia; 2 Research Fellow, Centre for Health Economics, Monash University, 900, Dandenong Road, Caulfield campus, Melbourne, VIC 3145 Australia

**Keywords:** Child growth, Midday meals programs, Plantation community, Sri Lanka, Weight-for-age, Height-for-age, Weight-for-height, Stunting, Underweight, Wasting

## Abstract

**Background:**

High rates of child malnutrition are a major public health concern in developing countries, particularly among vulnerable communities. Midday meals programs can be effective for combatting childhood malnutrition among older children. However, their use in early childhood is not well documented, particularly within South Asia. Anthropometric measures and other socioeconomic data were collected for children below the age of 5 years living in selected Sri Lankan tea plantations, to assess the effectiveness of midday meals as a nutrition intervention for improving growth among young children.

**Methods:**

The study exploits a natural experiment whereby the provision of the midday meals program is exogenously determined at the plantation level, resulting in comparable treatment and control groups. Longitudinal data was collected on heights and weights of children, between 2013 and 2015. Standardized weight-for-age, height-for-age and weight-for-height, and binary variables for stunting, wasting and underweight are constructed, following WHO guidelines. All modelling uses STATA SE 15. Random-effects regression with instrumental variables is used for modelling standardized growth while random-effects logistic regression is used for the binary outcomes. Robustness analysis involves different estimation methods and subsamples.

**Results:**

The dataset comprises of longitudinal data from a total of 1279 children across three tea plantations in Sri Lanka, with 799 children in the treatment group and 480 in the control group. Results show significant positive effects of access to the midday meals program, on the growth of children. A child with access to the midday meals intervention reports an average standardized weight-for-age 0.03 (±0.01) and height-for-age 0.05 (±0.01) units higher than a similar child without access to the intervention. Importantly, access to the intervention reduces the likelihood of being underweight by 0.45 and the likelihood of wasting by 0.47. The results are robust to different model specifications and across different subsamples by gender, birthweight and birth-year cohort.

**Conclusions:**

Midday meals programs targeting early childhood can be an effective intervention to address high rates of child malnutrition, particularly among vulnerable communities in developing countries like Sri Lanka.

## Background

Child malnutrition and mortality are key health issues in many developing countries. According to UNICEF/WHO/World Bank joint estimates, in 2018, 149 million children below the age of 5 were stunted while over 49 million children were wasted. More than half of all stunted children live in Asia [1]. Since the signing of the Millennium Declaration in 2000 Sri Lanka has made good progress in reducing child and maternal mortality. However child morbidity remains a key concern [2, 3]. Official statistics on child malnutrition also indicate significant regional variations at sector and district levels [2, 4–6].

The tea plantation community in Sri Lanka suffers from a long history of discrimination, neglect and deprivation [7]. Resident plantation systems were introduced to Sri Lanka in the mid-nineteenth century, during the British colonial period. Indian Tamil laborers were brought to the country from South India, to live and work within tea and rubber plantations. Treated as outsiders to the country, experiencing significant language barriers and geographic isolation, these Indian Tamil plantation communities faced significant social and economic disadvantage [8–10]. After years of political struggle, plantation communities of Indian descent were granted citizenship rights in the early 1990’s. Today, plantation communities represent 5% of the Sri Lankan population [11] and are recognized as a third sector, separate from the traditional urban and rural sectors in Sri Lanka. Existing literature points to poverty, poor health, malnutrition, lack of education opportunities and alcoholism as the major issues faced by a majority of the plantation community [12]. According to the 2016 Demographic and Health Survey (DHS), over 30% of children living within the plantation sector suffered from stunting while close to 30% were also underweight, prevalence far higher than the national averages of 17 and 20% respectively [2]. Children below the age of 5 years are particularly vulnerable to malnutrition given that the most crucial growth in humans occur within this period [13]. Malnourished children can grow up to be less productive adults, contributing to a continuing cycle of chronic poverty. It thus becomes imperative to disrupt the cycle of malnutrition within the early years of life.

The plantation sector of Sri Lanka has attracted many intervention programs aimed at improving living standards [14, 15]. Some well-known nutrition interventions targeting children are the *Thriposha* nutrition supplementation program, school milk program and the school midday meals program. *Thriposha* program which provides a protein-based cereal to children above the age of 6 months, but most other nutrition interventions target children of school-going age [16, 17]. This study considers the possibility of using midday meals programs as an effective mechanism for combating early childhood malnutrition. We exploit a natural experiment which relies on the staggered roll-out of a midday meals program (targeting children below the age of 5 years) across a number of tea plantations. We use longitudinal data to assess the impact of access to this program.

The midday meals program studied here is run by a major charitable foundation which focuses on plantation communities in Sri Lanka. Initiated in 2008, the midday meals program has been systematically introduced to tea plantations belonging to two major regional plantation companies (RPCs). Whilst RPCs account for around 25% of total tea/rubber plantation workers in Sri Lanka, these workers and their families are considered to have better living conditions and facilities than most, due to stronger corporate oversight of these companies. This suggests that any potential improvements achieved through the targeted intervention within this sub-population could potentially be even greater within the broader, more disadvantaged plantation community.

The midday meals program is implemented through child development centres (CDC) in tea estates. Given the female-intensive labour structure of the tea industry, these centres were introduced to provide childcare services to laborers who work in the plantations and have children below school-going age. The midday meals program was designed to provide a balanced daily midday meal to children at these centres. Registration at child development centres is limited to plantation employees. There is consistently high demand for manual labour within the industry, so there are few barriers to entry to this kind of employment, and hence to accessing the program.

This paper contributes to two main strands of the public health literature. First, given the popularity of midday meals as a nutrition intervention among school-going children, we explore the potential for its use in early childhood, which has not attracted much research especially in South Asia. This is a very important area for research, given the importance of the early years in child development, and the comparatively high rates of early childhood malnutrition in South Asia. Secondly, as these types of interventions are more widely adopted across developing countries, it is important to investigate their effectiveness in improving nutritional and growth outcomes of children in vulnerable settings, such as those living within Sri Lanka’s tea plantations. The evidence suggests that there is plenty of scope for expanding midday meals programs among pre-school aged children, and that a well-designed program with low barriers to entry is well suited to children in vulnerable communities.

## Methods

### Study setting and design

The sampling frame comprises of tea plantations belonging to one regional plantation company (RPC). The RPC managed a total of 12 tea plantations, five of which ran the midday meals program as of June 2015. The program was planned to be rolled out across the other seven plantations in due course. All plantations were located in close proximity within the Thalawakale region in Sri Lanka. This setup can be considered as a natural experiment where children from plantations with and without the midday meals program are compared. Longitudinal data was collected on the heights and weights of all children below the age of 5 years living in sampled plantations. Available demographic data was also collected for the sampled children together with institutional level data. Sample sizes required for the treatment and control groups were calculated following standard methodology for sample size determination in field surveys [18, 19]. The formula, $N=\frac{p\left(100-p\right){z}^2}{E^2}$ is used where *E* is set at 5%, *z* at 95 and 50% coverage of the program within the sampling frame is assumed. Simple Random Sampling using the Order Sampling approach was used to pick the sample of treatment and control plantations [20]. The sampling procedure resulted in 3 plantations (2 treatment and 1 control) being selected with a sample size of 799 children in the treatment group and 480 in the control group. The quantitative survey was conducted in June 2015.

The main source of anthropometric data and program attendance data was through midwife and CDC records. Consent for the provision of this data was obtained from plantation midwives and the child development officers (CDO) who head each CDC. Midwives and CDOs were also interviewed in order to collect institutional level data, information on the general health status of plantation children and details of the implementation and management of the midday meals programs. A housing quality checklist questionnaire was used to assess the general housing quality in each of the visited plantations, using a convenience sample of households.

### Ethics approval and data quality procedures

The necessary ethics approvals were obtained from the Monash University Human Research Ethics Committee (MUHREC) and the Plantations Human Development Trust - the main government authority overseeing human development within the tea industry in Sri Lanka. Approvals were also obtained from the management of the charitable foundation implementing the midday meals program and the regional plantation company managing the plantations.

As participants, written informed consent was obtained from all midwives and CDOs interviewed. Parental consent was not required by MUHREC or by the Sri Lankan authorities, as children were not directly interviewed during the survey. The study relies on data routinely collected and maintained by midwives and CDOs for health monitoring purposes. Complying with their approval and privacy protocols, this data on children’s weight and height measures was released by them in an appropriate non-identifiable format.

### Data

Anthropometric data for all children below the age of 5, living in sampled plantations was collected for the period January 2013–June 2015. Data comprised of an unbalanced panel of body weights (*kg*) and heights (*cm*) for children recorded by the estate midwife or CDOs. Weights were recorded monthly while the heights were recorded quarterly. Since data was only available for the period January 2013–June 2015, early-age weight and height measures for older children (3–5 year olds as of 2015) were not available. Each child’s panel started with the first available weight measure for the child. Possible impacts of this truncation of sample data is investigated as part of the robustness analysis. To match the monthly frequency of weight measures, missing monthly heights were imputed using cubic spline interpolation. Children’s weight-for-height/length was calculated following the conventional method [21].

Z-scores for the weight-for-age (*WAZ*), height-for-age (*HAZ*), and weight-for-height/length (*WHZ*) were obtained following WHO methodology [22], using their *igrowup* macros. Following WHO definitions for underweight, stunting and wasting [23], binary variables were then constructed to indicate if a child was underweight, stunted or wasted in any given time period, by considering whether *WAZ*, *HAZ* and *WHZ* for each child in each time period falls below − 2.00 respectively. These binary variables, together with the z-scores, are used as dependent variables in models.

Given the primary intention of the study is to provide insights that have relevance to other similar programs, it is important to use the globally accepted WHO methodology when constructing the dependent variables in the models [21]. There are a couple of relevant control variables where it is more appropriate to standardise using sample medians and standard deviations, as their purpose is to control for within-sample differences between children prior to the treatment. These are the standardized first weight/height measures for each child and their birthweight.

Other child level variables collected included date-of-birth, gender, birthweight and ethnic group. A CDC level variable indicating the physical condition of the CDC (old, upgraded or new) is also included in models. The treatment variable used in modelling is a time-invariant binary variable (*Trt*) identifying whether a child lived in a treatment or control plantation. Hence *Trt* identifies access to the midday meals program.

### Empirical strategy

Identification of the treatment effect relies on the staggered introduction of the midday meals program across similar tea plantations in similar localities and under the same management. The broad assumption here is that the plantations belonging to the treatment and control groups are generally similar – an assumption whose validity will be critically evaluated. Validity is also supported by relying on random selection of the specific plantations to include in the sample, from the populations of treatment and control plantations.

The following model is used to model the treatment effect:

1 $$ {H}_{i,t}=\alpha +{\beta}_1{Trt}_i+{\beta}_2{X}_{i,t}+{\beta}_3{Z}_i+{\varepsilon}_{i,t} $$

The subscripts *i* and *t* refer to child *i* at age *t* (in months). *H* represents a set of dependent variables measuring child growth - the standardized weight-for-age (*WAZ*), height-for-age (*HAZ*), weight-for-height/length (*WHZ*), and binary variables *underweight*, *stunting* and *wasting*. The Vectors *X* and *Z* include time-varying and time-invariant child-level variables respectively, while *Trt* is a binary variable indicating whether a child belonged to the treatment or control group. We assume all children in a treatment plantation have access to the midday meals program irrespective of the employment status of their parents/guardian. This assumption is deemed valid given the low barriers to entry to plantation employment throughout the year. Models for *WAZ*, *HAZ* and *WHZ* are fitted using a random-effects instrumental variable specification while models for *underweight*, *stunting* and *wasting* are fitted using random-effects logistic specification. All models fit robust standard errors.

Different variants of the above model were also fitted, to establish the robustness of the results. These include modelling by gender, birthweight and birth-year cohort, also helping explore equity issues, and using different estimation strategies (e.g. Generalized Least Squares Random Effects).

The limited number of observed demographic variables could mean there is a risk of omitted variable bias. Whilst a fixed-effects specification could have dealt with the issue of unobserved child-level variables, the time-invariant treatment variable does not allow for the use of fixed-effects. Therefore, it is important to address a number of specific concerns regarding the identification strategy.

#### Possible endogeneity of treatment variable

The treatment has been allocated across the tea plantations within the sampling frame based on managerial decisions about the order of roll-out of the program across plantations. Management report no systematic factors that influenced the decision about order, other than practical / administrative matters. However, if there are systematic unobserved differences between the treatment and control groups that are correlated with child development outcomes, this risks creating an endogeneity bias in model estimates. Baseline data can be used to check for similarity between treatment and control plantations. Since the midday meals intervention applies to children aged at least 6 months, we compare weight records of children up to 6 months of age across the treatment and control samples. Any differences cannot be attributed to the program, and instead could indicate relevant difference between the two groups.

**Fig. 1:**
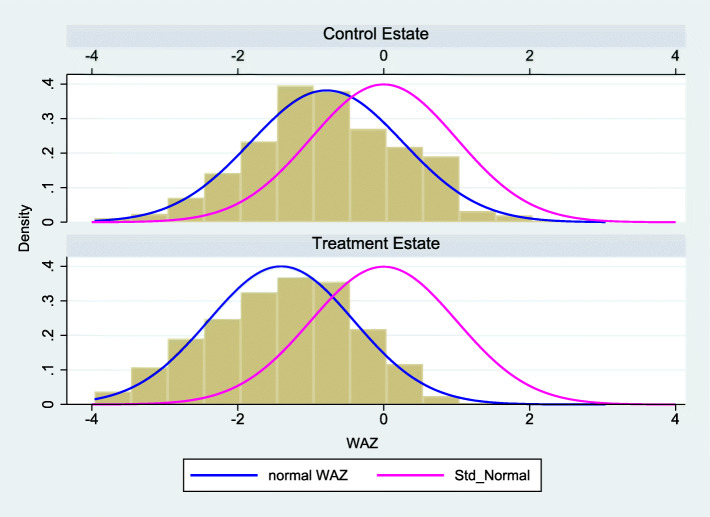
Histogram of weight-for-age zscores in the 0–6 month age category in treatment and control groups

The histograms of weight-for-age zscores (*WAZ*) in the 0–6 month age category across the treatment and control groups, together with the standard normal distribution curve, are presented in Fig. 1. According to this, the distribution of *WAZ* appear to be closer to the standard normal distribution within the control group than the treatment group. The two-sample Kolmogorov-Smirnov test for equality of distribution functions, rejects the null hypothesis of equality in distribution between the treatment and control groups (Combined K-S: 0.22, *p* < 0.000). These results suggest that children in the treatment group may be starting at a relative growth disadvantage compared to children in the control group, at the baseline.

A number of methods are used to overcome potential endogeneity of the treatment variable, the most significant of which is to control for measures of past growth, using a lagged dependent variable (*H*_*i*, *t* − 1_ in eq. (1)) as a control variable in the models. We also use other measures of past growth (birthweight, first recorded weight and height) as control variables. Proxy variables are also included to control for important unobserved time-invariant factors, where applicable. Together these measures should account for any significant time-invariant factors that may drive systematic differences between the treatment and control samples with respect to the growth of children. In addition to this we also include proxy variables to control for possible time-varying factors which could also cause systematic differences between the treatment and control groups. Added together, these methods provide reasonable confidence that we are capturing a causal effect with the treatment variable, having overcome any possible significant bias in our identification strategy. We next give details of the main relevant unobserved time-invariant and time-varying factors whose impacts are accounted for through this analytical approach.

#### Time-invariant unobserved variables

The first characteristics to consider are the general living standards and facilities in the estates. Systematic differences in these characteristics across treatment and control groups could, if not measured, find their way into the model error, and be correlated with the Treatment indicator variable, resulting in an endogenous treatment variable. In addressing this concern, a housing quality checklist was used to collect data on the average quality of housing within each plantation, and Principal Components [22] was used to construct a housing quality index. The housing quality index was statistically significant and positive in the weight-for-age model but not significant in height-for-age and weight-for-height models. Importantly, including this index in the model had virtually no impact on the estimated treatment effects.

Household income and food security are also not observed in our data, and these could also vary with the treatment and outcome variables, leading to an endogeneity risk. With regards to household income, most plantation families are employed within the tea industry [8, 24]. Daily wages paid to plantation workers are well regulated, consistently low and stagnant over time [8], and there is limited access to other forms of income through non-plantation work [8, 24]. This suggests that the average household income would not vary much over time and across the three study plantations. Given the stagnant nature of income over a period of just a few years, using past measures of growth (i.e. the prior period’s growth, first weight/height measure on record) would account for any time-invariant impact of household income on the growth of children.

We also consider food security as another possibly important unobservable. There is likely to be a strong link between household income and food security [25], so the arguments above regarding income would also apply to food security. The exception is the possibility that socio-cultural factors could also impact food security due to culturally induced food preferences or aversions. However, given that over 80% of plantation sector residents are from an Indian-Tamil ethnic background [24], it is reasonable to assume homogeneity in food preferences across the sampled plantations.

Family economic background and parental education are also unobserved household level variables which tend to be fixed over time [26]. Therefore, it is reasonable to assume that whilst these variables would impact child growth, they would not cause any systematic differences between treatment and control groups. Nevertheless, it is important to control for as many of these effects as possible. Birthweight is an ideal of a relevant proxy for the general socio-economic background of children as it impacts their growth. Apart from the rare occasion of a genetic/medical complication which impacts a child’s in utero growth, birthweight is usually a consequence of the nutrients that the mother receives during pregnancy. Past studies have established the close relationship between maternal and child nutrition, including in the plantation sector of Sri Lanka [27]. Therefore, we use birthweight as a proxy for the general economic background of the child’s family, especially around the time of birth.

#### Potential time-varying unobserved variables

The level of parental care and general child-care practices at home can have a significant impact on the growth of children, particularly considering the female dominant labour structure and the relatively large household sizes in tea plantations [25]. Even though there is little evidence to suggest that parental care would differ significantly between treatment and control plantations, not accounting for parental care received by the child may potentially bias our results. Therefore, it is important to control for this effect. We do so with a proxy based on accessing maternal health services. In Sri Lanka, parents of children living in identified vulnerable communities are strongly advised to visit a midwife for check-ups on a monthly basis. Regularity of monthly midwife visits would provide a proxy for the level of parental/guardian care and attention received by the child. A mother who attends all or most check-ups is likely to have a higher level of commitment to parental care than one who has erratic attendances. The proxy variable *pcare*_*i t*_ comprises the proportion of midwife/clinic visits completed compared to the number available, for each child *i* in each time period (months) *t*.

#### Inter-plantation migration due to midday meals program

A final methodological issue is the potential for plantation families to relocate between treatment and control plantations, based on the availability of the midday meals program. If this occurs, it is a possible source of bias in estimating treatment effects. In practice, this is highly unlikely, given the generally landless status of plantation residents [24]. Plantation residents are provided housing and other amenities through the plantation management and their right to the homes they live in is partly determined by several generations of a family living and working within the same plantation [8, 24]. There is virtually no freedom to relocate between plantations.

#### Sample truncation

Given the time window in which anthropometric measures were collected (i.e. January 2013–June 2015), non-random sample truncation is a possible concern in the study, as the window of observation does not allow for observing early measures of weights and heights for older children in the sample. We include the child’s birth year and age as independent variables in order to account for this effect. Further robustness tests are also carried out to determine the effects of non-random truncation/ attrition using sample attrition weights in models [28].

### Statistical analysis

De-identified height/weight data provided by the midwives and CDOs were entered to Microsoft Excel 2013 together with other child and CDC level variables. Data was then imported to STATA SE 15 and the dependent variables were constructed using the *WHO igrowup* macro.

#### Instrumental variables

The model in eq. (1) is fitted to standardized weight-for-age (*WAZ*), height-for-age (*HAZ*) and weight-for-height/length (*WHZ*). As indicated, past measures of growth (i.e. lagged *WAZ*, *HAZ* and *WHZ* etc.) are used as controls, to account for some of the unobserved time-invariant factors such as household income and food security. However, in *WAZ*, *HAZ* and *WHZ* models, this poses an additional analytical issue, as the lagged dependent variable becomes an intermediate variable in the causal pathway between the treatment and dependent variables. An intermediate variable can be defined as a variable which forms part of the causal pathway in the relationship between a treatment and an outcome. As a result, part of the treatment effect may be masked by the intermediate variable. In addition to this, there may also be other unobserved variables which impact both the present and previous period’s growth which could also bias results. For these reasons, we use an Instrument Variable Random Effects (IV RE) estimation method which instruments for the lagged dependent variable in *WAZ*, *HAZ* and *WHZ* models.

The instrumenting strategy involves a first-stage equation of the form:

2 $$ {H}_{i,t-1}={\delta}_1+{\delta}_2 IV+{\delta}_3{X}_{i,t}+{\delta}_4{Z}_i+{u}_{i,t} $$

where *IV* refers to the set of instruments used in each model and the vectors *X* and *Z* represent the time variant and time invariant variables included in eq. (1). *H* represents the lagged *WAZ*, *HAZ* or *WHZ*. Birthweight and the lagged parental care variable (*pcare*_*i t-1*_) are used to instrument for *WAZ*_*i*,t-1_ in eq. (2). Given the sample comprises of children below the age of 5 years, it is reasonable to assume that the birthweight would be correlated with the *WAZ* in early years, an assumption confirmed by the analysis. It is also reasonable to assume that the relationship between birthweight and *WAZ*_*i*,t_ will occur via *WAZ*_*i*,t-1_. Therefore, birthweight can be considered as a valid instrument for *WAZ*_*i*,t-1_ in the model.

Considering the lagged parental care variable (*pcare*_*i t-1*_), this variable reflects the general history of care received by children. Therefore, *pcare*_*i t-1*_ will also be strongly correlated with *WAZ*_*i*,t-1_. In addition to this it is again reasonable to assume that the relationship between the lagged parental care variable (*pcare*_*i t-1*_) and the current periods weight-for-age will manifest through the previous months weight-for-age. Therefore, *pcare*_*i t-1*_ can also be considered as a valid instrument for the lagged dependent variable in the weight-for-age model.

With regards to the height-for-age models, *pcare*_*i t-1*_ is again used as a valid instrument for *HAZ*_*i*,t-1_. A standardized value of the first height on record (*firstzheight*) is also used as an instrument. Birthweight, standardized value of first height (*firstzheight*) and the lagged parental care variable are all used as instruments for the previous period’s weight-for-height/length (*WHZ*_*i*,t-1_) in the weight-for-height/length models.

The validity of the instruments is assessed using the Kleibergen-Paap rk test (for under-identification), Cragg-Donald Wald F Test (weak instrument) and Sargan-Hansen test (validity of over-identification restrictions). Test results indicate that the instruments are valid in most of the main models. However, the validity of instruments cannot be confirmed in some of the subsample models, likely due to smaller sample sizes. Generalized Least Squares Random Effects (GLS RE) models are fitted in these cases, as a robustness test.

For modelling the binary variables *underweight*, *stunting* and *wasting* a Logit Random Effects estimation is used together with lagged values of *WAZ*, *HAZ* and *WHZ* as control variables respectively. An instrument variable approach is not necessary in this case as these control variables are not direct intermediate variables in these models.

## Results

### Descriptive analysis

Table 1 provides a descriptive summary of the dependent and control variables used in models. The overall sample was balanced with respect to gender: females (51%) and males (49%), and there is no statistically significant difference in the gender-split of the treatment and control groups. Mean birthweight in the overall sample is 2.5 kg, while the mean birthweight is slightly higher in the treatment group than the control group (difference is statistically significant). The percentage of low birthweight births is 39.4% in the overall sample, and not statistically different across the treatment and control groups. Looking across the dependent variables, the mean standardized weight-for-age (*WAZ*), height-for-age (*HAZ*) and weight-for-height/length (*WHZ*) are all negative in the overall sample, which is further evidence of the generally low growth status of plantation children. Mixed patterns are observed when comparing growth across treatment and control samples, with the treatment group showing better *HAZ* than the control group and the opposite pattern observed for *WAZ* and *WHZ*. Similar patterns are also observed with respect to the prevalence of underweight, wasting and stunting, with the percentage of wasting and stunting lower within the treatment group, compared to the control group. The mixed patterns further highlight the need for robust modelling in order to identify patterns in growth differences across treatment and control groups.

**Table 1:** Descriptive statistics of the main dependent and control variables

	Total			Treatment			Control			*P*-value^1^
	N	Mean/Prop	95% CI	N	Mean/Prop	95% CI	N	Mean/Prop	95% CI	
**Dependent V**										
*WAZ*	17,214	−1.69	(−1.70, − 1.67)	11,834	−1.74	(− 1.76, − 1.72)	5380	−1.58	(− 1.60, − 1.55)	0.0000
*HAZ*	10,309	−2.01	(− 2.03, − 1.99)	7581	− 1.94	(− 1.97, − 1.92)	2728	−2.20	(− 2.24, − 2.15)	0.0000
*WHZ*	7783	−0.88	(−0.91, − 0.86)	5939	− 0.96	(− 0.99, − 0.94)	1844	−0.62	(− 0.68, − 0.55)	0.0000
*underweight* (Base: Yes)	17,214	36.8%	(36.1%, 37.5%)	11,834	38.3%	(37.5%, 39.2%)	5380	33.4%	(32.1%, 34.7%)	0.0000
*wasting* (Base: Yes)	7783	14.9%	(14.1%, 15.7%)	5939	14.7%	(13.8%, 15.6%)	1844	15.4%	(13.8%, 17.2%)	0.4605
*stunting* (Base: Yes)	10,309	50.1%	(49.1%, 51.0%)	7581	47.3%	(46.1%, 48.4%)	2728	57.8%	(56.0%, 59.7%)	0.0000
**Control V**										
Parental care	17,214	86.6%	(86.4%, 86.9%)	11,834	85.4%	(85.1%, 85.6%)	5380	89.4%	(89.0%, 89.8%)	0.0000
Birthweight (kg)	1148	2.50	(2.47, 2.54)	735	2.54	(2.50, 2.57)	413	2.44	(2.39, 2.50)	0.0061
Low Birthweight (Base: Yes)	1148	39.4%	(36.5%, 42.3%)	735	37.7%	(34.2%, 41.3%)	413	42.4%	(37.6%, 47.3%)	0.1189
Gender (Base: Male)	1279	49.3%	(46.6%, 52.1%)	799	47.8%	(44.3%, 51.3%)	480	51.9%	(47.3%, 56.4%)	0.1591
Ethnicity (Base: Sinhala)	1279	8.7%	(7.2%, 10.4%)	799	10.9%	(8.8%, 13.3%)	480	5.0%	(3.2%, 7.3%)	0.0003

^1^ *P*-value column indicates the *p*-value for the 2-tailed test which compares the respective variable between the Treatment and Control groups

### Baseline growth indicators across treatment and control groups

As noted in Fig. 1, the treatment and control groups show significant differences in baseline levels of weight-for-age. In this section, we look at the treatment and control groups in more detail. Table 2 provides a comparison of the percentage of *underweight*, *wasting* and *stunting* in the overall sample, 0–6 month and 7–60 month age categories. Children in the treatment groups appear to have relatively poor growth measures compared to the control group at the baseline. A similar pattern is also evident with the percentage of *wasting*, where a relatively higher percentage is observed within the 0–6 months age category within the treatment group. This pattern however, is not observed with regards to *stunting* at the baseline. Comparing with the 7–60 month age category, there is an obvious increase in the percentage of underweight and wasted children compared to the 0–6 month age group, and that the increase is greater for the control group. Stunting rates increase with age for both the treatment and control groups, with similar magnitudes. The worsening of child growth with age, compared to WHO reference populations, is a phenomenon often detected in developing countries [29]. However, the results suggest that while children in the treatment group may be starting at a relative growth disadvantage compared to children in the control group, the impact of the midday meals program appears to be helping bridge this early growth gap between the two groups and somewhat slow down the decline in relative growth with age within the treatment group. More comprehensive models to follow will seek to confirm this observation.

**Table 2:** Prevalence of underweight, wasting and stunting by age category and group

	Overall [Prop (95% CI)]		Treatment [Mean (95% CI)]		Control [Mean (95% CI)]	
	0–6 mon	7–60 mon	0–6 mon	7–60 mon	0–6 mon	7–60 mon
*underweight*	23.2% (21.0%, 25.0%)	38.0% (37.3%, 38.8%)	29.2% (26.3%, 32.3%)	39.1% (38.2%, 40.0%)	12.3% (9.5%, 15.5%)	35.6% (34.2%, 36.9%)
N	1422	15,792	917	10,917	505	4875
*wasting*	3.8% (0.8%, 10.7%)	15.0% (14.2%, 15.8%)	11.1% (0.3%, 48.2%)	14.3% (13.8%, 15.6%)	2.9% (0.3%, 9.9%)	15.9% (14.3%, 17.7%)
N^a^	79	7704	9	5930	70	1774
*stunting*	46.8% (36.4%, 57.4%)	50.1% (49.1%, 51.1%)	33.3% (7.5%, 70.1%)	47.3% (46.2%, 48.4%)	48.2% (37.3%, 59.3%)	58.1% (56.2%, 60.0%)
N^a^	94	10,215	9	7572	85	2643

^a^Sample size for wasting and stunting in the 0–6 month age category is small due to the limited number of height/length records available for children in this age group and used in models

### Main treatment effects

**Fig. 2:**
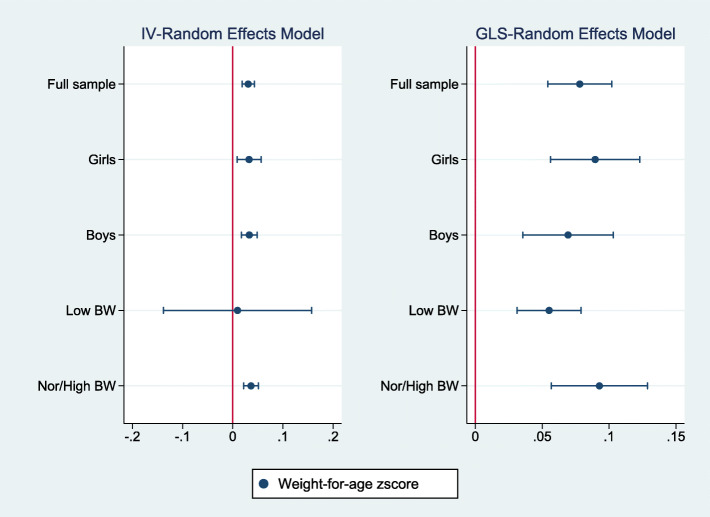
The relationship between weight-for-age zscore and access to the midday meals program^2^. ^2^ Figures 2, 3, 4 Notes: Regressions are estimated using robust standard errors. The treatment variable is a binary variable equal to one if the child lives in a plantation which runs the midday meals interventions. Dots denote the point estimates for the treatment effect and horizontal lines denote the 95% confidence intervals. The left panel shows the results based on instrument-variable random effects (IV-RE) estimation, while the right panel shows the results using generalized least squares random-effects (GLS-RE). The vertical red line represents the zero-reference line

**Fig. 3:**
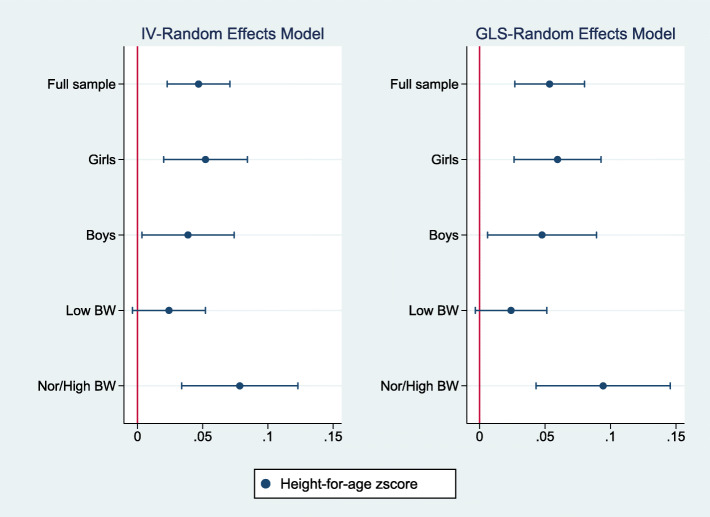
The relationship between height-for-age zscore and access to the midday meals program

**Fig. 4:**
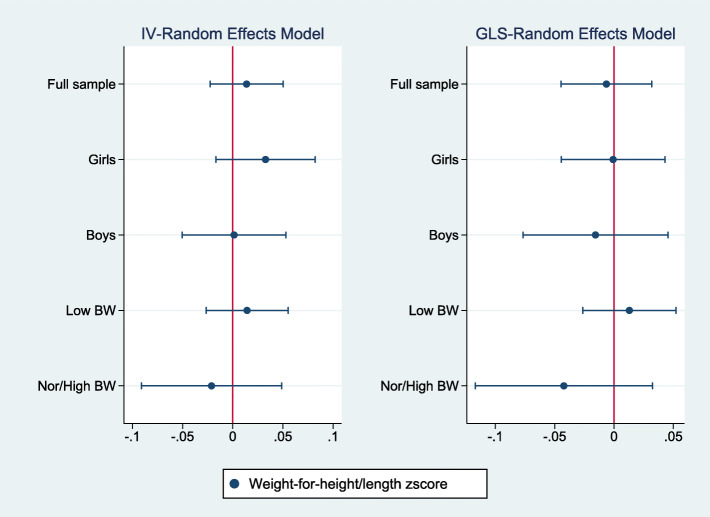
The relationship between weight-for-height/length zscore and access to the midday meals program

Figures 2, 3, 4 summarize the results of a series of regressions depicting the relationship between the growth status (*WAZ*, *HAZ* and *WHZ*) of children and access to the midday meals intervention whilst Tables 3, 4, 5 provide the numerical results for the estimated treatment effects depicted in Figs. 2, 3 and 4 respectively.

**Table 3:** Weight-for-age zscore vs access to the midday meals program- IV-RE and GLS-RE results

	(1)	(2)	(3)	(4)	(5)
	Full sample	Girls	Boys	Low BW	Normal/High BW
Panel A: IV-RE regressions					
Treatment var. (Trt)	0.031** (0.006)	0.033** (0.012)	0.033** (0.008)	0.009 (0.075)	0.036** (0.007)
Other controls?	Yes	Yes	Yes	Yes	Yes
Panel B: GLS-RE regressions					
Treatment var. (Trt)	0.078** (0.012)	0.090** (0.017)	0.069** (0.017)	0.055** (0.012)	0.093** (0.018)
Other controls?	Yes	Yes	Yes	Yes	Yes
Observations	17,214	8567	8647	7336	9878

Notes: Robust standard errors in parenthesis, **p < 0.01, *p < 0.05, + *p* < 0.1

The dependent variable is weight-for-age zscore. Estimated treatment effects using the full sample, girls, boys, low birthweight and normal/high birthweight cohorts presented. The treatment variable (Trt) is a dummy equal to one if the child had access to the midday meals program. Panel A provides results for the IV-Random Effects model and Panel B provides results for GLS-Random Effects Model. Other controls include child age, age squared, gender, zscore of first weight, pcare, zscore of birthweight, ethnicity, CDC status and birth-year fixed effects either as direct controls or instruments

**Table 4:** Height-for-age zscore vs access to the midday meals program- IV-RE and GLS-RE results

	(1)	(2)	(3)	(4)	(5)
	Full sample	Girls	Boys	Low BW	Normal/High BW
Panel A: IV-RE regressions					
Treatment var. (Trt)	0.047** (0.012)	0.052** (0.016)	0.039* (0.018)	0.024+ (0.014)	0.078** (0.023)
Other controls?	Yes	Yes	Yes	Yes	Yes
Panel B: GLS-RE regressions					
Treatment var. (Trt)	0.053** (0.014)	0.059** (0.017)	0.048* (0.021)	0.024+ (0.014)	0.094** (0.026)
Other controls?	Yes	Yes	Yes	Yes	Yes
Observations	10,309	5285	5024	4847	5462

Notes: Robust standard errors in parenthesis, **p < 0.01, *p < 0.05, + *p* < 0.1

The dependent variable is height-for-age zscore. Estimated treatment effects using the full sample, girls, boys, low birthweight and normal/high birthweight cohorts presented. The treatment variable (Trt) is a dummy equal to one if the child had access to the midday meals program. Panel A provides results for the IV-Random Effects model and Panel B provides results for GLS-Random Effects Model. Other controls include child age, age squared, gender, zscore of first weight, zscore of birthweight, zscore of first height, pcare, ethnicity, CDC status and birth-year fixed effects either as direct controls or instruments

**Table 5:** Weight-for-height/length zscore vs access to the midday meals program- IV-RE and GLS-RE results

	(1)	(2)	(3)	(4)	(5)
	Full sample	Girls	Boys	Low BW	Normal/High BW
Panel A: IV-RE regressions					
Treatment var. (Trt)	0.014 (0.018)	0.033 (0.025)	0.001 (0.026)	0.014 (0.021)	−0.021 (0.036)
Other controls?	Yes	Yes	Yes	Yes	Yes
Panel B: GLS-RE regressions					
Treatment var. (Trt)	−0.006 (0.019)	− 0.001 (0.022)	− 0.016 (0.031)	0.013 (0.020)	− 0.042 (0.038)
Other controls?	Yes	Yes	Yes	Yes	Yes
Observations	7783	3943	3840	3748	4035

Notes: Robust standard errors in parenthesis, **p < 0.01, *p < 0.05, + *p* < 0.1

The dependent variable is weight-for-height zscore. Estimated treatment effects using the full sample, girls, boys, low birthweight and normal/high birthweight cohorts presented. The treatment variable (Trt) is a dummy equal to one if the child had access to the midday meals program. Panel A provides results for the IV-Random Effects model and Panel B provides results for GLS-Random Effects Model. Other controls include child age, age squared, gender, zscore of first weight, zscore of birthweight, zscore of first height, pcare, ethnicity, CDC status and birth-year fixed effects either as direct controls or instruments

In Tables 3, 4, 5, columns (1), (2), (3), (4) and (5) provide estimates for the full sample, among girls, boys, children with low birthweight and children with normal/high birthweight respectively. Panel A provides the estimated effects for the IV-Random Effects regression model while Panel B presents estimated results for the GLS-Random Effects model. It should be noted that where IV models are valid, estimates using GLS models are likely to be higher as they do not correct for the intermediation effect of the lagged dependent variable. Regressions include all controls discussed above and robust standard errors.

Figures 2, 3, 4 report estimates of the coefficients of the Treatment variable, along with 95% confidence intervals denoted by the vertical lines either side of the point estimates. The left panel shows results using instrument-variable random effects (IV-RE) estimation, while the right panel shows results using generalized least squares random-effects (GLS-RE). From the top, the panels depict results for the full sample and subsamples of girls, boys, low birthweight children and normal/high birthweight children respectively. The vertical line in red represents the zero-reference line, indicating no treatment effect.

Table 3 below presents the estimated treatment effects on the weight-for-age zscore while Fig. 2 presents the same results graphically. The results show that having access to the midday meals intervention is associated with a statistically significant increase in the weight-for-age zscores, in the full sample, among girls, among boys and among children having a normal/high birthweight. Estimated effects vary between 0.03 and 0.04 standard deviation (SD) units in IV-RE models and between 0.05 to 0.09 SD units in the GLS-RE models. The treatment effect is not statistically significant among children having low birthweight in the IV-RE model. However, the instrument validity tests report the IV-RE model to be underidentified in the low birthweight cohort, implying that the GLS-RE estimate would be more reliable in this subsample. This indicates a statistically significant treatment effect in the low birthweight cohort. However, the magnitude of the treatment effect is clearly smaller compared to the normal/high birthweight cohort. Similar effects are observed across the other samples suggesting that the magnitude of the effect of the intervention on the weight-for-age zscore is similar across the different cohorts, with the possible exception of the low birthweight cohort.

Table 4 and Fig. 3 presents the estimated treatment effects for the height-for-age zscore. Again, access to the midday meals intervention is associated with a statistically significant increase in the height-for-age zscores, in the full sample, among girls, among boys and among children having a normal/high birthweight. Only marginally significant treatment effects are observed among children having low birthweight (significance at 10%). The estimated effects range between 0.02 and 0.08 SD across the IV-RE models and between 0.02 to 0.09 SD units in the GLS-RE models. The magnitude of the treatment effect is also higher among girls compared to boys.

Table 5 and Fig. 4 presents the estimated effects of access to the midday meals intervention on the weight-for-height/length of children. The point estimates are not statistically significant in the full sample and across the different cohorts. This suggests that access to the midday meals intervention does not show significant impacts on the weight-for-height/length of children.

Table 6 presents results for weight-for-age, height-for-age and weight-for-height models using the full sample. Regressions include all controls including the child’s birth year.

**Table 6:** IV and GLS regression results - full model

		*IV and GLS – random effects regression*					
		*WAZ*		*HAZ*		*WHZ*	
		IV	GLS	IV	GLS	IV	GLS
Treatment var							
*Trt*		0.031** (0.006)	0.078** (0.012)	0.047** (0.012)	0.053** (0.014)	0.014 (0.018)	-0.006 (0.019)
Instrumented var							
*WAZ*_*i,t-1*_/ *HAZ*_*i,t-1*_/ *WHZ*_*i,t-1*_		1.012** (0.025)	0.731** (0.009)	0.913** (0.008)	0.904** (0.011)	0.819** (0.023)	0.814** (0.011)
Child-level							
*Age*		0.006** (0.002)	-0.005** (0.001)	0.007** (0.002)	0.007** (0.002)	-0.008* (0.003)	-0.008* (0.003)
*Age_sq*		-0.0001** (0.00002)	0.0001** (0.00002)	-0.0001** (0.00003)	-0.0001* (0.00003)	0.0001+ (0.00004)	0.0001+ (0.00004)
*Gender*	Male	0.016** (0.004)	-0.012 (0.011)	-0.004 (0.01)	-0.005 (0.011)	-0.007 (0.014)	-0.009 (0.015)
*firstzweight*		-0.043* (0.017)	0.14** (0.009)	0.037** (0.006)	0.038** (0.006)	0.082** (0.013)	0.076** (0.009)
*firstzheight*					0.006 (0.01)		0.004 (0.009)
*pcare*		0.07** (0.019)	0.077* (0.033)	0.074* (0.036)	0.093* (0.043)	-0.080 (0.059)	-0.048 (0.059)
*zbirthweight*			0.02** (0.005)	-0.004 (0.005)	-0.004 (0.005)		0.020* (0.008)
*Ethnicity*	Sinhalese	0.004 (0.007)	0.012 (0.022)	0.019 (0.018)	0.019 (0.019)	-0.070** (0.025)	-0.063** (0.024)
CDC-level							
*CDC_category*	New	0.004 (0.006)	-0.002 (0.017)	-0.002 (0.015)	-0.004 (0.016)	-0.040+ (0.022)	-0.052* (0.022)
	Upgraded	0.004 (0.006)	0.0005 (0.015)	0.008 (0.016)	0.011 (0.017)	-0.028 (0.021)	-0.034 (0.021)
_cons		-0.111 (0.091)	-0.348** (0.096)	-0.577** (0.081)	-0.626** (0.089)	0.363** (0.130)	0.342** (0.133)
Observations		17214	17214	10309	10309	7783	7783
*σ*_*u*_		0.035	0.094	0.108	0.153	0	0.098
*σ*_*e*_		0.309	0.306	0.237	0.224	1.222	0.460
*ρ*		0.012	0.086	0.171	0.318	0	0.044
*Kleibergen-Paap LM Stat*		23.80**		123.44**		61.96**	
*Cragg-Donald Wald F Stat*		82.13		1262.49		330.80	
*Sargan-Hansen Test*		0.613		0.138		9.328**	

Robust standard errors in parenthesis, **p<0.01, *p<0.05, + *p*<0.1

K-P LM test [Null: matrix of reduced form coefficients has rank=K1-1 (underidentification)], C-D Wald F Test [Null: Equation is weakly identified], S-H Test [Null: Overidentification restriction is satisfied]. Birth-year fixed effects excluded from table

Table 7 and Table 8 present IV-RE model results by gender and birthweight cohorts respectively. For simplicity, tables present only the estimated treatment effects and instrument validity explained below. Estimated effects of other controls are mostly similar to those presented in Table 6 above.

**Table 7:** IV regression model results- by *Gender*

	*IV– Random Effects Regression*					
	*Female*			*Male*		
	*WAZ*	*HAZ*	*WHZ*	*WAZ*	*HAZ*	*WHZ*
Treatment var						
*Trt*	0.033** (0.012)	0.052** (0.016)	0.033 (0.025)	0.033** (0.008)	0.039* (0.018)	0.001 (0.026)
Other controls?	Yes	Yes	Yes	Yes	Yes	Yes
Observations	8567	5285	3943	8647	5024	3840
*σ*_*u*_	0.039	0.090	0	0.060	0.109	0
*σ*_*e*_	0.299	0.335	1.225	0.350	0.243	1.136
*ρ*	0.017	0.068	0	0.029	0.167	0
*Kleibergen-Paap LM Stat*	9.28**	68.84**	38.25**	15.85**	65.35**	34.55**
*Cragg-Donald Wald F Stat*	31.95	689.29	118.05	37.61	785.13	230.37
*Sargan-Hansen Test*	0.457	0.666	8.162*	0.132	0.019	5.473+

Robust standard errors in parenthesis, **p < 0.01, *p < 0.05, + *p* < 0.1

K-P LM test [Null: matrix of reduced form coefficients has rank = K1–1 (underidentification)], C-D Wald F Test [Null: Equation is weakly identified], S-H Test [Null: Overidentification restriction is satisfied]. Other controls include child age, age squared, gender, zscore of first weight, zscore of birthweight, zscore of first height, pcare, ethnicity, CDC status and birth-year fixed effects either as direct controls or instruments

**Table 8:** IV regression model results- by *Birthweight*

	*IV– Random Effects Regression*					
	*Low BW*			*Normal/High BW*		
	*WAZ*	*HAZ*	*WHZ*	*WAZ*	*HAZ*	*WHZ*
Treatment var						
*Trt*	0.009 (0.075)	0.024+ (0.014)	0.014 (0.021)	0.036** (0.007)	0.078** (0.023)	−0.021 (0.036)
Other controls?	Yes	Yes	Yes	Yes	Yes	Yes
Observations	7336	4847	3748	9878	5462	4035
*σ*_*u*_	0	0.102	0	0	0.114	0
*σ*_*e*_	15.420	0.227	1.457	0.430	0.246	0.873
*ρ*	0	0.168	0	0	0.176	0
*Kleibergen-Paap LM Stat*	0.14	64.62**	27.54**	25.88**	56.42**	33.97**
*Cragg-Donald Wald F Stat*	0.44	687.65	184.18	106.09	548.89	159.33
*Sargan-Hansen Test*	0.668	0.080	0.406	4.136*	0.059	2.018

Robust standard errors in parenthesis, **p < 0.01, *p < 0.05, + *p* < 0.1

K-P LM test [Null: matrix of reduced form coefficients has rank = K1–1 (underidentification)], C-D Wald F Test [Null: Equation is weakly identified], S-H Test [Null: Overidentification restriction is satisfied]. Other controls include child age, age squared, gender, zscore of first weight, zscore of birthweight, zscore of first height, pcare, ethnicity, CDC status and birth-year fixed effects either as direct controls or instruments

### Validity of instruments

The Kleibergen-Paap LM, Cragg-Donald and the Sargan-Hansen (S-H) tests (for underidentification, weak-identification and overidentification respectively) were carried out to check the validity of the instruments used in the IV models. All three tests confirm the validity of the instruments used in the weight-for-age and height-for-age models fitted to the full sample. The S-H score indicates that the overidentification restriction is rejected for the weight-for-height model. In that case, the GLS model estimates are also considered, and the observed treatment effect is not significant in either case. For models fitted in subsamples by gender and birthweight, the instruments were found to be valid in almost all cases.

### Treatment effects by birth-year cohort

In the plantations belonging to the treatment group, the midday meals intervention was initiated in 2007. This implies that children born between 2007 and 2009, within the treatment group, would have been the first batch of children to have access to the intervention from early infancy whilst, children born between 2010 and 2012, would have been beneficiaries of a relatively more mature midday meals program. Children born between 2013 and 2015 would have had access to a well-seasoned program. It is interesting to explore possible differential effects for children within these three birth-year cohorts.

**Fig. 5:**
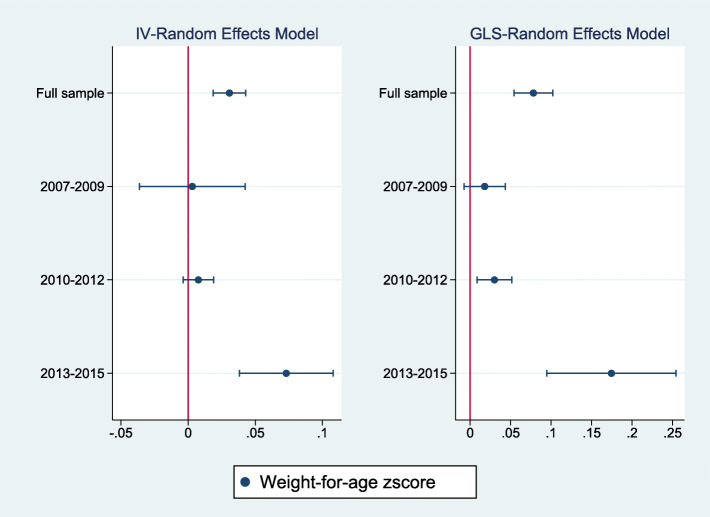
The relationship between weight-for-age zscore by birth-year cohort and access to the midday meals program^3^. ^.3^ Figures 5, 6, 7 Notes: Regressions are estimated using robust standard errors. The treatment variable is a binary variable equal to one if the child lives in a plantation which runs the midday meals interventions. Dots denote the point estimates for the treatment effect and horizontal lines denote the 95% confidence intervals. The left panel shows the results based on instrument-variable random effects (IV-RE) estimation, while the right panel shows the results using generalized least squares random-effects (GLS-RE). The vertical line represents the zero-reference line

**Fig. 6:**
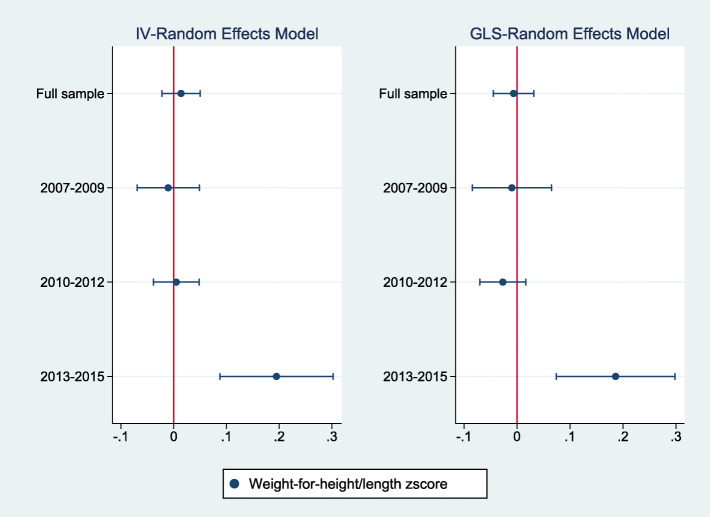
The relationship between weight-for-height/length zscore by birth-year cohort and access to the midday meals program

**Fig. 7:**
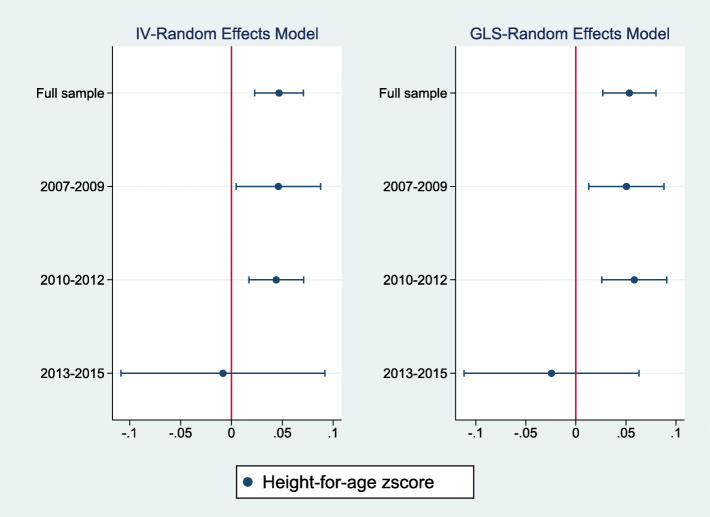
The relationship between height-for-age zscore by birth-year cohort and access to the midday meals program

Tables 9, 10, 11 provide treatment effects for the weight-for-age, weight-for-height/length and height-for-age models respectively. Columns (1), (2), (3) and (4) provide estimates for the full sample, among 2007–2009, 2010–2012 and 2013–2015 birth-year cohorts. Panel A provides the estimated effects for the IV-Random Effects regression model while Panel B presents estimated results for the GLS-Random Effects model. Figures 5, 6, 7 present the same results graphically.

**Table 9:** Weight-for-age zscore vs access to the midday meals program: by birth-year cohort

	(1)	(2)	(3)	(4)
	Full sample	2007–2009	2010–2012	2013–2015
Panel A: IV-RE regressions				
Treatment var. (Trt)	0.031** (0.006)	0.003 (0.020)	0.008 (0.006)	0.073** (0.018)
Other controls?	Yes	Yes	Yes	Yes
Panel B: GLS-RE regressions				
Treatment var. (Trt)	0.078** (0.012)	0.018 (0.013)	0.030** (0.011)	0.174** (0.041)
Other controls?	Yes	Yes	Yes	Yes
Observations	17,214	2468	11,192	3554

Notes: Robust standard errors in parenthesis, **p < 0.01, *p < 0.05, + *p* < 0.1

The dependent variable is weight-for-age zscore. Estimated treatment effects from regressions using the full sample, 2007–2009, 2010–2012 and 2013–2015 birth cohorts presented. The treatment variable (Trt) is a dummy equal to one if the child had access to the midday meals program. Panel A provides results for the IV-Random Effects model and Panel B provides results for GLS-Random Effects Model. Other controls include child age, age squared, gender, zscore of first weight, pcare, zscore of birthweight, ethnicity, CDC status and birth-year fixed effects either as direct controls or instruments

**Table 10:** Weight-for-height/length zscore vs access to the midday meals program: by birth-year cohort

	(1)	(2)	(3)	(4)
	Full sample	2007–2009	2010–2012	2013–2015
Panel A: IV-RE regressions				
Treatment var. (Trt)	0.014 (0.018)	−0.011 (0.030)	0.005 (0.022)	0.195** (0.055)
Other controls?	Yes	Yes	Yes	Yes
Panel B: GLS-RE regressions				
Treatment var. (Trt)	− 0.006 (0.019)	−0.010 (0.038)	− 0.027 (0.022)	0.186** (0.057)
Other controls?	Yes	Yes	Yes	Yes
Observations	7783	1281	5835	667

Notes: Robust standard errors in parenthesis, **p < 0.01, *p < 0.05, + *p* < 0.1

The dependent variable is weight-for-height zscore. Estimated treatment effects from regressions using the full sample, 2007–2009, 2010–2012 and 2013–2015 birth cohorts presented. The treatment variable (Trt) is a dummy equal to one if the child had access to the midday meals program. Panel A provides results for the IV-Random Effects model and Panel B provides results for GLS-Random Effects Model. Other controls include child age, age squared, gender, zscore of first weight, zscore of birthweight, zscore of first height, pcare, ethnicity, CDC status and birth-year fixed effects either as direct controls or instruments

**Table 11:** Height-for-age zscore vs access to the midday meals program: by birth-year cohort

	(1)	(2)	(3)	(4)
	Full sample	2007–2009	2010–2012	2013–2015
Panel A: IV-RE regressions				
Treatment var. (Trt)	0.047** (0.012)	0.046* (0.021)	0.044** (0.014)	−0.008 (0.051)
Other controls?	Yes	Yes	Yes	Yes
Panel B: GLS-RE regressions				
Treatment var. (Trt)	0.053** (0.014)	0.050** (0.019)	0.058** (0.017)	−0.024 (0.045)
Other controls?	Yes	Yes	Yes	Yes
Observations	10,309	1878	7562	869

Notes: Robust standard errors in parenthesis, **p < 0.01, *p < 0.05, + *p* < 0.1

The dependent variable is height-for-age zscore. Estimated treatment effects from regressions using the full sample, 2007–2009, 2010–2012 and 2013–2015 birth cohorts presented. The treatment variable (Trt) is a dummy equal to one if the child had access to the midday meals program. Panel A provides results for the IV-Random Effects model and Panel B provides results for GLS-Random Effects Model. Other controls include child age, age squared, gender, zscore of first weight, zscore of birthweight, zscore of first height, pcare, ethnicity, CDC status and birth-year fixed effects either as direct controls or instruments

First, consider the estimated treatment effects on the weight-for-age zscore (Table 9 and Fig. 5). Statistically significant treatment effects are only observed in the 2013–2015 birth-year cohort where access to the midday meals intervention is associated with an increase of 0.07 SD on average, in the weight-for-age zscore. The intervention also has a statistically significant positive effect on the weight-for-height/length of children within the 2013–2015 birth-year cohort as indicated in Table 10 and Fig. 6, where the intervention is associated with an increase of 0.19 SD in the weight-for-height zscore.

An opposite pattern is observed in the height-for-age models presented in Table 11 and Fig. 7 where the estimated treatment effects are statistically significant for the two older birth-year cohorts 2007–2009 and 2010–2012. Access to the midday meals intervention is associated with an average increase of 0.05 SD and 0.04 SD in the height-for-age zscore among children born between 2007 and 2009 and 2010–2012 respectively.

These results suggest that, as intended, the program has a positive influence on improving the height-for-age among children who have had a longer period of involvement, implying that the program is effective in improving long-term growth. The positive influence on improving weight would presumably, in time translate into improvements in height.

With regards to the validity of instruments used within the subsamples, the K-P, C-D and S-H tests report the instruments to be valid in all fitted models, with the exception of three models (underidentification in *WAZ* model (2007–2009) and overidentified in *WHZ* model (2010–2012) and *HAZ* model (2013–2015)). However, the treatment effects are not statistically significant for both the IV and GLS models in all three instances noted. Table 12 presents the estimated treatment effects and instrument validity results for the IV-RE models for the birth-year cohorts.

**Table 12:** IV regression model results- by *Birth-Year Cohort*

	*IV– Random Effects Regression*								
	*2007–2009*			*2010–2012*			*2013–2015*		
	*WAZ*	*HAZ*	*WHZ*	*WAZ*	*HAZ*	*WHZ*	*WAZ*	*HAZ*	*WHZ*
Treatment var									
*Trt*	0.003 (0.020)	0.046* (0.021)	−0.011 (0.030)	0.008 (0.006)	0.044** (0.014)	0.005 (0.022)	0.073** (0.018)	−0.008 (0.051)	0.195** (0.055)
Other controls?	Yes	Yes	Yes	Yes	Yes	Yes	Yes	Yes	Yes
Observations	2468	1878	1281	11,192	7562	5835	3554	869	667
*σ*_*u*_	0	0	0	0	0.098	0	0	0.142	0
*σ*_*e*_	1.438	2.180	0.615	0.401	0.274	0.892	0.756	0.389	0.763
*ρ*	0	0	0	0	0.113	0	0	0.118	0
*Kleibergen-Paap LM Stat*	0.27	31.08**	25.97**	8.73*	91.40**	36.65**	20.72**	17.80**	15.88**
*Cragg-Donald Wald F Stat*	0.72	458.22	194.30	43.68	858.58	180.00	114.15	360.20	60.80
*Sargan-Hansen Test*	2.036	0.081	0.116	0.040	1.227	11.552**	1.194	4.262*	3.522

Robust standard errors in parenthesis, **p < 0.01, *p < 0.05, + *p* < 0.1

K-P LM test [Null: matrix of reduced form coefficients has rank = K1–1 (underidentification)], C-D Wald F Test [Null: Equation is weakly identified], S-H Test [Null: Overidentification restriction is satisfied]. Other controls include child age, age squared, gender, zscore of first weight, zscore of birthweight, zscore of first height, pcare, ethnicity, CDC status and birth-year fixed effects either as direct controls or instruments

### Treatment effects on underweight, wasting and stunting

**Fig. 8:**
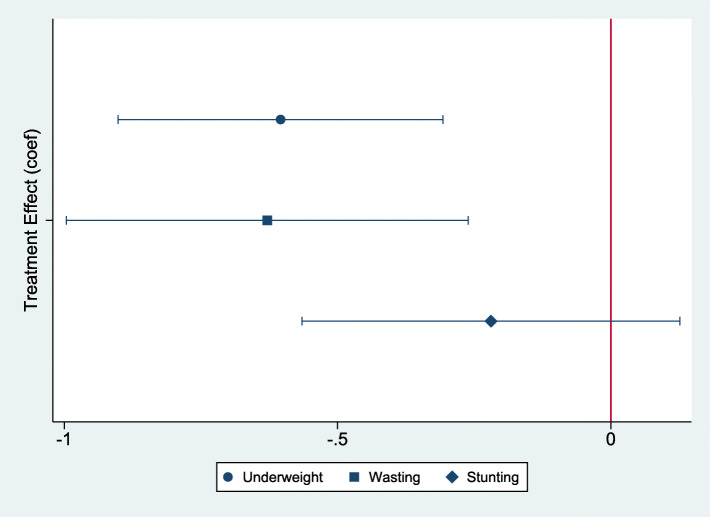
The relationship between the likelihood of being underweight, wasted and stunted and access to the midday meals program (coef)

Logistic regression models were fitted to model the relationship between access to the midday meals intervention and the likelihood of children being underweight, wasted and stunted. Figure 8 presents the estimated coefficients and Table 13 presents the estimated odds ratios and fractional odds of these adverse outcomes based on access to the intervention. The estimated coefficients are all negative, indicating that access to the intervention reduces the likelihood of being underweight, wasted and stunted. However, statistically significant effects of the treatment are detected only on the likelihood of being underweight and wasted. According to the fractional odds in Table 13, for a given child, the odds of being underweight and wasted decreased by 0.45 (45%) and 0.47 (47%) respectively, if the child had access to the midday meals intervention. The results clearly indicate the effectiveness of the midday meals intervention in reducing the prevalence of underweight and wasting. No statistically significant effects are observed on the prevalence of stunting.

**Table 13:** Estimated coefficients, odds-ratio and fractional odds for the treatment effect in underweight, wasting and stunting models

Model	Underweight	Wasting	Stunting
Coef (Std Dev)	−0.604**(0.152)	−0.628** (0.187)	−0.219 (0.176)
95% CI	(−0.901, −0.307)	(−0.996, − 0.261)	(− 0.565, 0.127)
Odds-Ratio (Std Dev)	0.547** (0.083)	0.533** (0.100)	0.803 (0.142)
95% CI	(0.406, 0.736)	(0.369, 0.770)	(0.568, 1.135)
Fractional Odds (%)	−0.453** (−45.3%)	− 0.467** (−46.7%)	−0.197 (−19.7%)
95% CI	(−0.594, − 0.264)	(−0.631, − 0.230)	(−0.432, 0.135)
Observations	17,214	7783	10,309

Robust standard errors in parenthesis, **p < 0.01, *p < 0.05, + *p* < 0.1

Results of the logistic random effects models fitted for the binary variables *underweight*, *wasting* and *stunting* are presented in Table 14. Table presents only the estimated treatment effect for simplicity.

**Table 14:** Logit RE regression – Underweight, Wasting and Stunting

	*Logit– Random Effects Regression*					
	*Underweight*		*Wasting*		*Stunting*	
	*Coef*	*Odds R*	*Coef*	*Odds R*	*Coef*	*Odds R*
Treatment var						
*Trt*	−0.604** (0.152)	0.547** (0.083)	−0.628** (0.187)	0.533** (0.100)	−0.219 (0.176)	0.803 (0.142)
Other controls?	Yes	Yes	Yes	Yes	Yes	Yes
Observations	17,214		7783		10,309	
ln*σ*_*u*_	0.490		−0.701		−0.308	
*σ*_*u*_	1.278		0.704		0.857	
*ρ*	0.332		0.131		0.183	

Robust standard errors in parenthesis, **p < 0.01, *p < 0.05, + *p* < 0.1. Other controls include lagged *WAZ*/*HAZ*/WHZ, child age, age squared, gender, zscore of first weight, zscore of birthweight, zscore of first height, pcare, ethnicity, CDC status and birth-year fixed effects

### Robustness

As noted earlier, non-random attrition poses a significant challenge given the time window in which anthropometric measures were collected. Following standard methods for accounting for attrition [28, 30], we re-estimate eq. (1) using attrition weights. The inclusion of attrition weights did not impact the statistical significance or direction of the treatment effects in the *WAZ* and *HAZ* models. The magnitude of the estimated effects is very similar but slightly lower, in line with expectations. Therefore, attrition is not considered to create a bias in the various estimates of treatment effects.

Other robustness checks included using different estimation methods (i.e. GLS-RE) and subsamples (by gender, birthweight and birth-year), results of which were previously discussed.

## Discussion

**Fig. 9:**
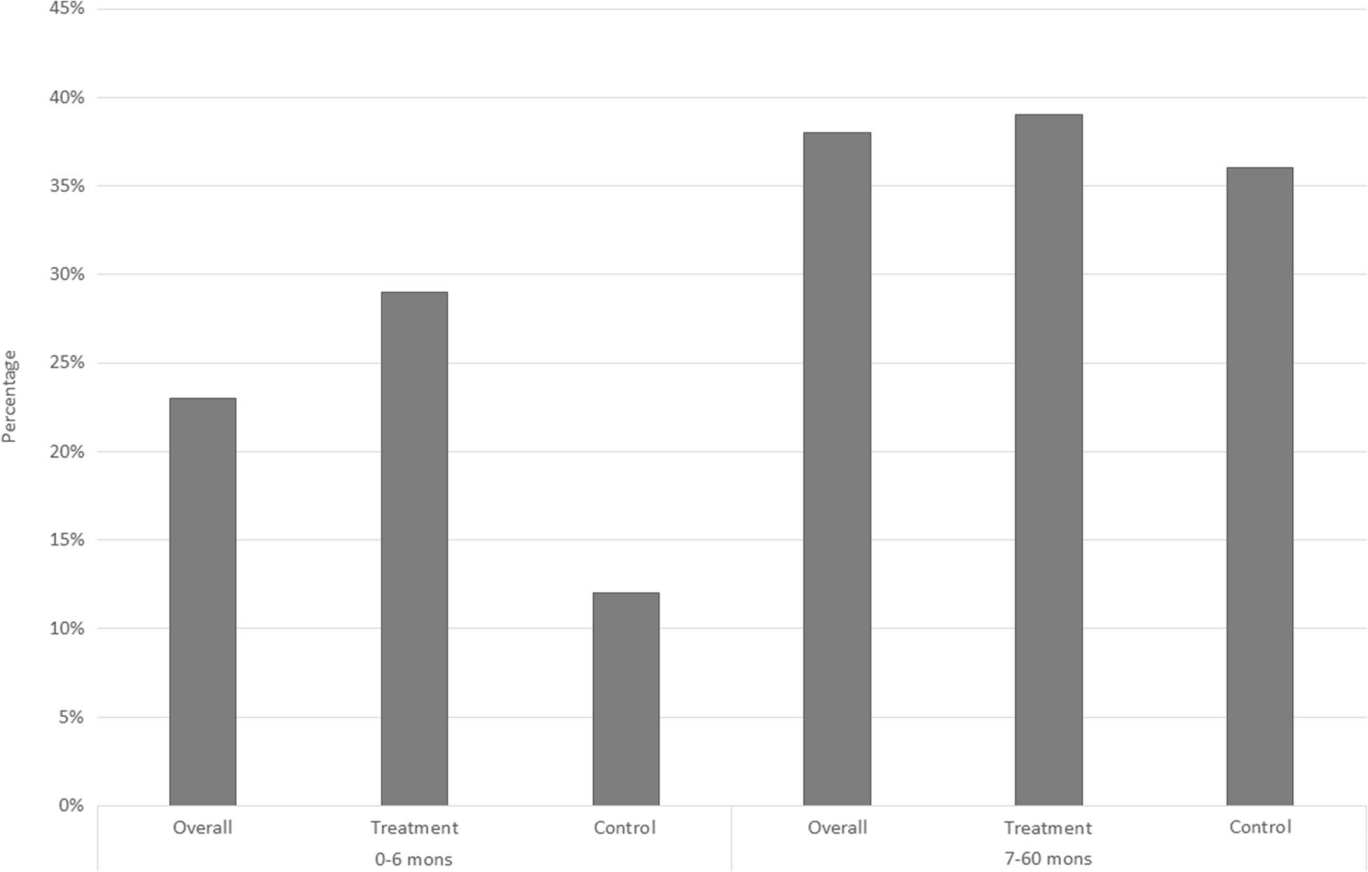
Percentage underweight within the 0–6 month and 7–60 month age category within the overall, treatment and control samples

**Fig. 10:**
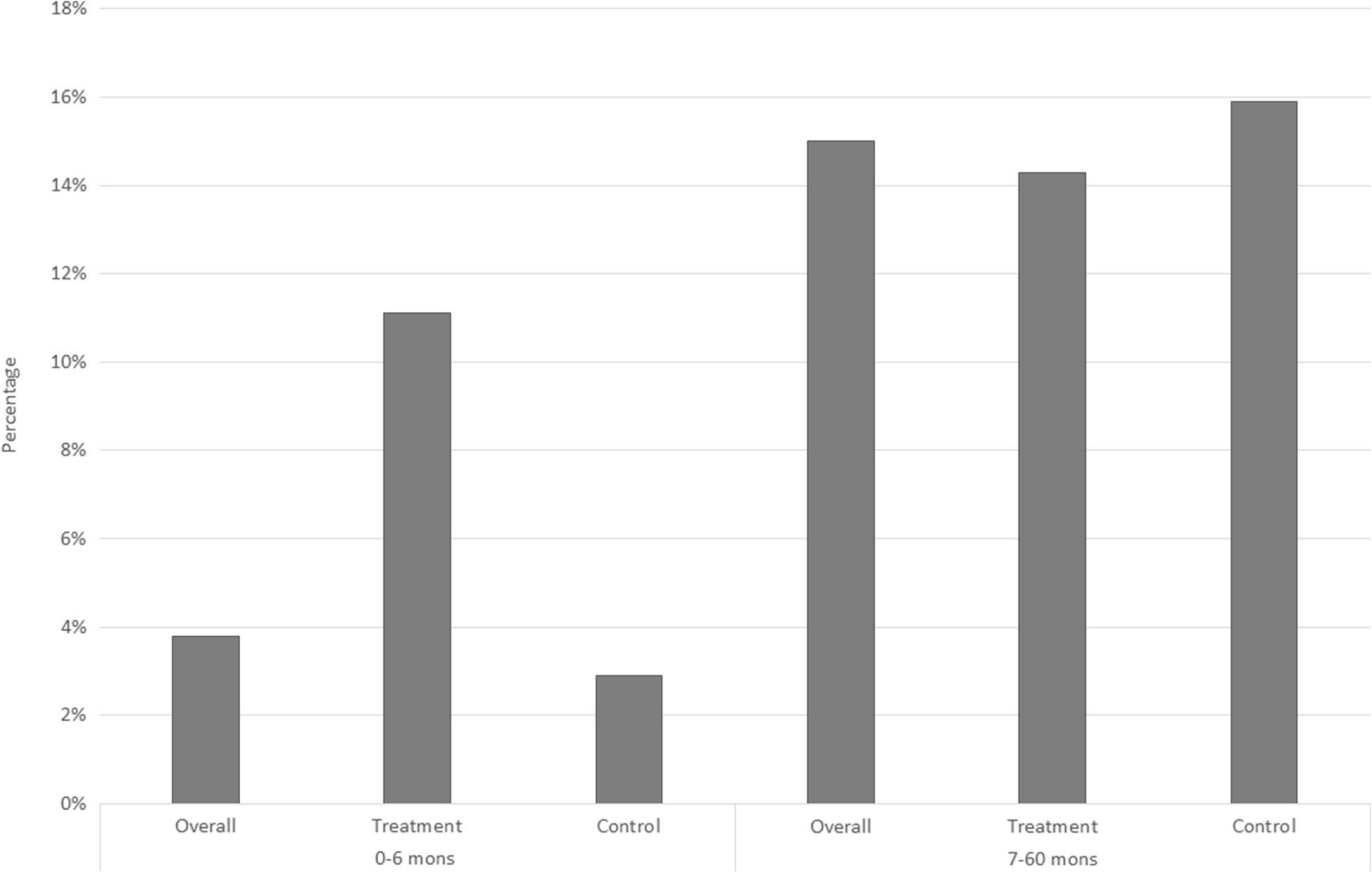
Percentage wasting within the 0–6 month and 7–60 month age category within the overall, treatment and control samples

**Fig. 11:**
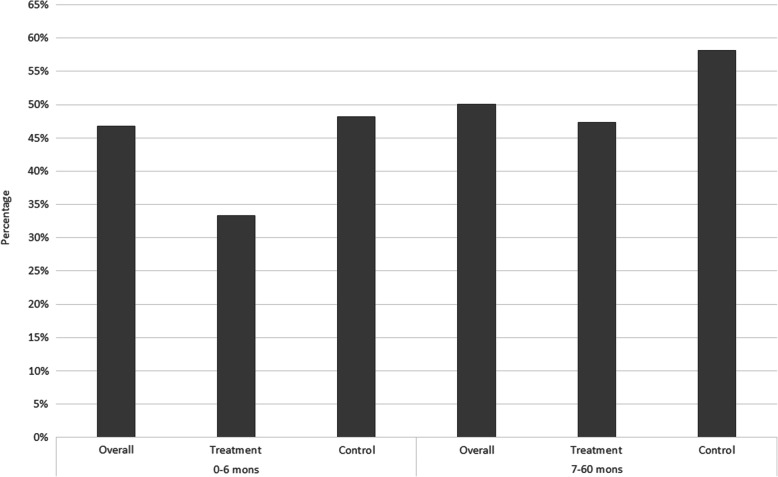
Percentage stunting within the 0–6 month and 7–60 month age category within the overall, treatment and control samples

The primary intention of this paper was to identify whether access to the midday meals program had a positive impact on the growth of pre-school children living within tea plantation communities. Overall results clearly indicate a strong positive impact of access to the midday meals intervention on the growth of children at different levels. Preliminary analysis revealed that the treatment group displayed relatively poor anthropometric measures compared to the control group prior to joining the treatment (age below 6 months). However, growth clearly improved within the treatment group over the treatment period (7–60 months age group). Figures 9, 10, 11 present the information provided in Table 2 in a different form. The figures in order, present the percentage of underweight, wasted and stunted children in the overall, treatment and control samples in the 0–6 month and 7–60 month age groups. A few patterns clearly emerge. The percentage of underweight and wasting has clearly increased in the overall, treatment and control samples in the 7–60 month age category compared to the 0–6 month age category. In Fig. 9, the percentage of underweight children in the control group increases substantially from the 0–6 month to 7–60 month age categories, whilst the increase is far smaller in the treatment group. Figure 10 shows a similar pattern where the percentage of wasting shows a much larger increase of approximately 13% from the 0–6 month to 7–60 month age category within the control group. The treatment group experience a much smaller increase in the percentage of wasting of approximately 3%. With regards to the percentage of stunting in Fig. 11, an increase in the percentage is observed from the baseline to the 7–60 month age category, across all three groups.

These results suggest two clear points. Firstly, results clearly indicate a general deterioration in growth for children living within the tea plantation community, compared to the WHO reference population. This is in line with similar patterns observed in other studies where the growth of children in developing countries is closer to the reference population at birth but falls further behind with age [29]. This deterioration in relative growth with age is smaller within the treatment group than the control group, particularly for underweight and wasting, suggesting clear beneficial impact of the midday meals program as a nutritional safety net.

Model estimates using the overall sample suggest that access to the midday meals program results in significantly improved standardized weight-for-age and height-for-age of children. Most importantly, the positive effects for height-for-age suggest that midday meals programs of this nature can be used as effective tools for improving child growth and for helping nutritionally deprived children to catch up in growth. Having access to a program such as the midday meals program, with low entry barriers, can act as a nutritional safety net for pre-school aged children. The logistic model results also indicate that access to the midday meals program significantly reduces the prevalence of underweight and wasting among children within tea plantations.

It is useful to examine the factors that may drive these positive effects. Discussions with midwives who regularly see the mothers and children, suggest significant issues related to accessing protein-rich food, intake of imbalanced meals and dietary preferences within the plantation communities, highlighting these factors as potential drivers of child malnutrition within tea plantations. Inappropriate feeding practices and lack of dietary diversity have also been highlighted in prior research into the nutrition status of plantation communities in Sri Lanka [31, 32].

Issues surrounding pregnancy patterns and antenatal care were also highlighted by midwives, particularly noting that plantation women tend to have 3–4 close pregnancies, resulting in a lack of care afforded to older children in many cases. This trend can also lead to significant challenges with food security at the household level. The midday meals intervention would be able to effectively address this issue, through the provision of at least one balanced daily meal to children. Multiple children from the same family being part of the midday meals program will also promote equitable access to nutrients among children belonging to the same family.

Alcohol abuse is a concern that has also been noted in several other studies [12, 32, 33]. This highlights the potential usefulness of programs such as the midday meals intervention in the setting of tea plantations in Sri Lanka, over interventions such as cash transfer programs, which give discretion to parents about how the cash is spent with possible adverse outcomes for children.

When considering the impact of the intervention within particular cohorts, the occasional greater benefits of program access for girls compared to boys is an interesting observation. A breakdown of actual program take-up by gender showed similar program take-up by girls and boys, which indicates that the observed differential effects are not driven by gender differences in treatment adoption or intensity. One possible explanation for this difference is that in the control group, boys are favoured in the rationing of food within the household, while boys and girls are treated more equally in the treatment group due to their access to the midday meal, where there is no gender difference in the provision of food. This pattern warrants further exploration through future studies.

Access to the midday meals intervention disproportionately favours children born with normal or high birthweights compared to children born with low birthweight. While a statistically significant positive treatment effect on the weight-for-age of low birthweight children was observed in some models, the magnitude of the effect was much lower for low birthweight children compared to children with normal/high birthweight. No statistically significant treatment effects were observed on the height-for-age of children in the low birthweight cohort. This may imply that the intervention falls short of benefiting the long-run growth of more vulnerable cohorts of children, who may require more targeted interventions. From a program design perspective, this is critical and warrants further exploration to identify why program benefits appeared to be weaker among the children who need it the most. It is notable that a breakdown of actual program take-up in the treatment plantations showed that only 35% of children born with low birthweight participated in the midday meals program in one of the treatment plantations, whilst a relatively higher percentage of low birthweight children (67%) were program participants in the other plantation. This could be the main reason for the non-significant treatment effects among low birthweight children and suggests a clear need for promoting and popularizing the midday meals program among families with low birthweight children. Additional support may also be required for these children (e.g. nutrition education, regular monitoring, and supplementary nutrition), to correct for early growth deficits.

With regards to the adaptability of midday meals interventions to other settings, there is a wide network of established child development centres (CDCs) within tea plantations in Sri Lanka where similar midday meals programs can be implemented. Previous studies have highlighted the impact of CDCs and the crucial role of child development officers (CDOs) in facilitating interactions between children, their mothers and public health midwives as well as their potential to act as mediators in child-centred development programs [31]. This makes CDCs and CDOs the ideal setting for a widescale implementation of a nutrition intervention such as a midday meals program. It is also common to maintain home-gardens within CDCs, encouraging community participation through the provision of fresh home-grown produce for preparation of the midday meals at CDCs. Together, these factors point towards the high feasibility and scalability of such programs within the target population.

To conclude this discussion, we note that while the paper has sent a clear message of the nutritional benefits of a midday meals program for vulnerable children aged 0–5 years, the study has left several questions unanswered about the mechanisms by which these benefits are produced. It is also acknowledged that while the sample size is reasonably large (1279 children), the study is based on children from just three (large) plantations, posing a risk about the generalisability of the findings. The research agenda would benefit from other studies that confirm the patterns found here. The findings reported to this point suggest that such research is well justified, in demonstrating the benefits of these programs for children.

## Conclusion

This analysis clearly suggests that midday meals can be used as an effective early childhood nutrition intervention to promote child growth among vulnerable population groups such as the tea plantation community in Sri Lanka. Midday meals programs can be expanded into child development centres in other tea plantations in Sri Lanka, as well as in similar centres that exist among other vulnerable communities. Extending the concept of these midday meals to pre-school aged children provides great hope for early intervention in dealing with the nutritional deficits so prevalent among children in the developing world.

## Data Availability

The datasets used and/or analysed and the Public Health Midwife Questionnaire, CDO Questionnaire and Household Quality Checklist used in the study are available from the corresponding author on reasonable request.

## Abbreviations

CDC: Child development centre
CDO: Child development officer
CI: Confidence interval
DHS: Demographic and health survey
GLS-RE: Generalized least squares random-effects
HH: Household
IV-RE: Instrument variable random-effects
MUDREC: Monash university human research ethics committee
OR: Odds ratio
RPC: Regional plantation company
WHO: World health organization
HAZ: Standardized height-for-age
WAZ: Standardized weight-for-age
WHZ: Standardized weight-for-height/length
